# Combination of the Level-Set Methods with the Contourlet Transform for the Segmentation of the IVUS Images

**DOI:** 10.1155/2012/439597

**Published:** 2012-05-22

**Authors:** Hassen Lazrag, Med Saber Naceur

**Affiliations:** ^1^Ecole Nationale d'Ingénieurs de Tunis (ENIT), B.P. 37, Le Belvedere, 1002 Tunis, Tunisia; ^2^Institut National des Sciences Appliquées et de Technologie (INSAT), Centre Urbain Nord, B.P. 676, 1080 Tunis, Tunisia

## Abstract

Intravascular ultrasound (IVUS) imaging is a catheter-based medical methodology establishing itself as a useful modality for studying atherosclerosis. The detection of lumen and media-adventitia boundaries in IVUS images constitutes an essential step towards the reliable quantitative diagnosis of atherosclerosis. In this paper, a novel scheme is proposed to automatically detect lumen and media-adventitia borders. This segmentation method is based on the level-set model and the contourlet multiresolution analysis. The contourlet transform decomposes the original image into low-pass components and band-pass directional bands. The circular hough transform (CHT) is adopted in low-pass bands to yield the initial lumen and media-adventitia contours. The anisotropic diffusion filtering is then used in band-pass subbands to suppress noise and preserve arterial edges. Finally, the curve evolution in the level-set functions is used to obtain final contours. The proposed method is experimentally evaluated via 20 simulated images and 30 real images from human coronary arteries. It is demonstrated that the mean distance error and the relative mean distance error have increased by 5.30 pixels and 7.45%, respectively, as compared with those of a recently traditional level-set model. These results reveal that the proposed method can automatically and accurately extract two vascular boundaries.

## 1. Introduction

Intravascular ultrasound (IVUS), one of the most recent developments in medical imaging, is a new method for coronary heart disease diagnosis and endovascular surgery. It renders real-time, cross-sectional and high-resolution images of blood vessels and provides information concerning the vascular lumen and wall. Besides, IVUS imaging permits visualization of atherosclerotic lesion morphology and precise measurements of arterial dimensions. However, a typical intravascular ultrasound pull-back sequence generally produces several hundreds of images, which has the effect of making nonautomatic analysis of the data long, fastidious and subject to high intra- and interobserver variability. These could be serious limitations against the medical usage of IVUS technique. In addition, because of poor IVUS image quality due to the existence of speckle, imaging artefacts, calcification shadowing, and rupture of parts of the arterial wall [1], it is necessary to develop efficient segmentation methods taking into account the nature of IVUS images.

Several approaches for automated segmentation have been proposed in the last decade for the analysis of IVUS images, including statistical texture analysis [2], knowledge-based searching [3], active contours [4], and minimum cost algorithms [5]. Among them, the active contour models [6, 7] showed remarkable feasibility and accuracy for the detection of lumen and media-adventitia boundaries in the IVUS. In the level-set models, an initial contour is first placed in an energy field of the image and then iteratively evolved to the desired curve and position by minimizing a certain energy functional. When variational level-sets are applied in the detection of lumen and media-adventitia borders of IVUS images, they will encounter two difficulties. First, to ensure the detection sensitivity and specificity, the two initial contours should be localized near the two desired boundaries. Second, after the two initial contours are appropriately determined, a robust algorithm should be developed to help level-set functions accurately deform to final borders; otherwise, level sets may be misled to undesired vascular borders because of the presence of the speckle noise and artifacts in IVUS images, including guide-wire artifact and ring-down artifact.

In this paper, we propose to combine the contourlet transform with the level-set curve evolution algorithm to overcome these two difficulties. The contourlet transform (CT) with its extra feature of directionality achieves better results than the discrete wavelet transform and yields new potentials in removing speckle noise and preserving the edge well without the effort to set a threshold. The CT is a new two-dimensional image transform, which provides a multiscale, local and multidirectional image representation using multiscale (Laplacian pyramid, LP) and directional filter banks (DFB) [8]. With the contourlet transform, an original image is decomposed into low-pass bands and band-pass bands. The low-pass components can be regarded as multiresolution set of images from coarse to fine, from which the two initial contours of the blood vessels can be extracted effectively by using the Hough transform [9, 10].

The band-pass components contain different information about the edge and speckle noise at multiple scales and directions [11]. The anisotropic diffusion (AD) filtering on these directional band-pass bands, as well as the level-set functions, composes a robust algorithm to evolve curves and obtain accurate final borders.

This paper is organized as follows.  Section 2.1.1 depicts about the theoretical background of level-set formulation. We briefly review the contourlet transform in Section 2.1.2. Our contourlet-based level set segmentation approach is described in Section 2.2. Experimental results are demonstrated and devoted to discussion in Session 3. Some essential conclusions are summarized in Section 4. In the following sections, the approach is validated using both the simulated and in vivo IVUS images. 

## 2. Methods

### 2.1. Theoretical Backgrounds

#### 2.1.1. Level-Set Formulation Model

In image segmentation, active contours [6, 7] are self-deforming dynamic curves or interfaces that move under the influence of internal and external forces, towards the object boundaries or image features which we want to extract. The segmentation model developed further is defined on level-set without reinitialization method (LSWR), and the adopted approach is inspired from [12]. Due to the new presented internal energy, the reinitialization procedure is completely eliminated. The evolution of each interface is guided by internal and external forces. The proposed level-set segmentation approach was directly derived from the *gradient flow* that minimizes the overall energy functional.

For a given image *I*, the desired contours are denoted by Γ(*t*). The level-set function *φ*(*x*, *y*, *t*) is Lipschitz continuous satisfying

(1) $$\begin{array}{cc} \varphi \left(x,y,t\right)<0, & \;\left(x,y\right)\;\text{is}\;\text{inside}\;\Gamma \left(t\right),\\ \varphi \left(x,y,t\right)=0, & \;\left(x,y\right)\;\text{is}\;\text{at}\;\Gamma \left(t\right),\\ \varphi \left(x,y,t\right)>0, & \;\left(x,y\right)\;\text{is}\;\text{outside}\;\Gamma \left(t\right). \end{array}$$

Hence, the region contours are completely described by the zero-level-set function *φ*(*x*, *y*, *t*); that is,

(2) Γ(t)=  {(x,  y,  t):φ(x,  y,  t)=0}.

To achieve this goal, the authors [12] define an external energy that can move the zero-level-set curve toward the image boundaries. This overall energy is defined as follows:

(3) $$E\left(\varphi \right)=\mu P\left(\varphi \right)+E_{m}\left(\varphi \right),$$

where *μ* > 0 is a parameter controlling the effect of penalizing the deviation of *φ* from assigned distance function. The internal energy *P* penalizes the deviation of *φ* from the signed distance during its evolution, while the external energy *E*_*m*_ derives the level-set function toward the image boundaries.

The internal energy is defined by the following integral:

(4) $$P\left(\varphi \right)=\int_{\Omega }{\left(\left|\nabla \varphi \right|-1\right)}^{2}dX,$$

as a metric to characterize how close a function *φ* is to a signed distance function in *Ω* ∈ *R* ^2^. This metric will play a key role in the proposed level-set segmentation model.

The external energy is defined for a function for a function *φ* as follows:

(5) $$E_{m}\left(\varphi \right)=\lambda L\left(\varphi \right)+\upsilon A\left(\varphi \right),$$

where *λ* > 0 and *υ* are constants, and the facts *L*(*φ*) and *A*(*φ*) are defined by

(6) $$\begin{array}{c} L\left(\phi \right)=\int_{\Omega }g\delta \left(\varphi \left(X\right)\right)\left|\nabla \varphi \left(X\right)\right|dX,\\ A\left(\phi \right)=\int_{\Omega }gH\left(\varphi \left(X\right)\right)dX, \end{array}$$

respectively, where *δ* is the Dirac function and *H* is the Heaviside function. For numerical stability of the delta function, lets define the classical regular approximations *H*_*α*_ and *δ*_*α*_ of the Dirac and Heaviside functions with *α* ∈ *ℜ* ^+^:

(7) $$\delta_{\alpha }\left(s\right)=\frac{1}{2\alpha }\left(1+\cos\frac{\pi s}{\alpha }\right),\;H_{\alpha }\left(s\right)=\frac{1}{2}\left(1+\frac{\text{s}}{\alpha }+\frac{1}{\pi }\sin\frac{\pi \text{s}}{\alpha }\right).$$

In order to guarantee the convergence of level-set curves on the set of boundaries, an edge indicator function *g* is used. This edge indicator function is defined for an image *I* by

(8) $$g\left(\nabla I\right)=\frac{1}{1+{\left|G_{\sigma }*\nabla I\right|}^{2}},$$

where *G*_*σ*_ is the Gaussian kernel with a standard deviation *σ*, ∗ is the convolution operator, and *I∇* is the gradient of the image *I*. To overcome the reinitialization process using in traditional level set, the authors [12] proposed a fast level-set formulation:

(9) $$\begin{array}{cc} \frac{\partial \varphi }{\partial t} & =\mu \left[\Delta \varphi -\mathrm{div}\left(\frac{\nabla \varphi }{\left|\nabla \varphi \right|}\right)\right]\\ & \begin{array}{c} \;+\lambda \delta \left(\varphi \right)\mathrm{div}\left(g\frac{\nabla \varphi }{\left|\nabla \varphi \right|}\right)+vg\delta \left(\varphi \right), \end{array} \end{array}$$

(10) $$\varphi_{0}\left(x,y\right)=\{\begin{array}{cc} -C, & \text{if}\;\left(x,y\right)\in R_{0}^{2},\\ C, & \text{otherwise}, \end{array}$$

where *C* is customable constant and *R*_0_ is a region in the domain *Ω*.

This gradient flow (9) with the initial function *φ*_0_ (10) is the evolution equation of the *LSWR* method, where as the second term attacks the level-set function *φ* towards the variational boundary. However, the first term is a penalty momentum of *φ*, which deviates from the signed distance function and has important advantages. First, the novel algorithm eliminates the computationally expensive reinitialization process. Second, it may start from an arbitrary initial contour *φ*_0_.

#### 2.1.2. Contourlet Transform

The Contourlet transform was developed by Do and Vetterli [8] to overcome the drawbacks of wavelets in two-dimensional domain. This transform not only possesses main features of wavelets (namely, multiresolution and spatial-frequency localization), but it also shows a high degree of directionality and anisotropy. The contourlet transform is useful to capture intrinsic geometrical structures such as smooth contours in images, including medical images. It is implemented by decomposing the original image into multiscale with 2D Laplacian pyramid (LP) and then decomposing subbands at each level into directional components with Directional Filter Bank (DFB). In this double filter bank, the LP first captures point discontinuities and then the DFB links point discontinuities into linear structures.

Specifically, let *a*_0_(*x*, *y*) be an input image. Then, the outputs after a one-level LP decomposition are a coarse approximation coefficient *a*_1_(*x*, *y*) and detail coefficient *b*_1_(*x*, *y*), representing the low-pass band and band-pass component, respectively. The approximation image *a*_1_(*x*, *y*) can be further decomposed into coarser image *a*_2_(*x*, *y*) and a detail image *b*_2_(*x*, *y*) by the LP, which are coefficients at level 2. The detail image *b*_1_(*x*, *y*) can be decomposed by *l*_1_-level DFB into 2^*l*_1_^ band-pass directional images *c*_1,*k*_^(*l*_1_)^, *k* = 0,1,…, 2^*l*_1_^ − 1. Similarly, after *J*-level LP decomposition, the low-pass bands *a*_1_(*x*, *y*), *a*_2_(*x*, *y*),…, *a*_*J*_(*x*, *y*) compose a sequence of multiresolution images from fine to coarse, and the band-pass directional components *c*_1,0_^(*l*_1_)^(*x*, *y*),…, *c*_1,2^*l*_1_^−1_^(*l*_1_)^(*x*, *y*),…, *c*_*J*,0_^(*l*_*J*_)^(*x*, *y*),…, *c*_*J*,2^*l*_*J*_^−1_^(*l*_*J*_)^(*x*, *y*) represent multiresolution and multidirectional signals.

**Figure 1:**
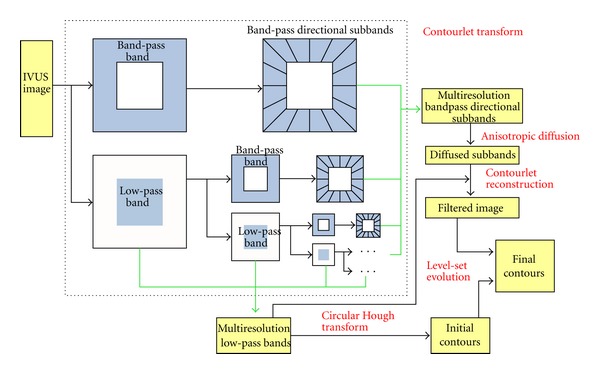
Main framework of contourlet level-set model (contourlet transform shown in dashed block).

The contourlet transform scheme is illustrated in the dashed block in Figure 1, where the input is an image and the output is low-pass bands and band-pass directional subbands.

### 2.2. Contourlet-Based Level-Set IVUS Image Segmentation Model (CLSM)

This paper presents a new level-set model that fully integrates the advantages of contourlet transform. The multiresolution low-pass components are used to yield the initial contours of lumen and media-adventitia borders. The band-pass directional subbands are utilized to extract the final contours. The main framework of the proposed model is shown in Figure 1.

#### 2.2.1. Initial Contour Extraction

In level-set segmentation model, it is difficult to extract the initial contours from image sequence. The Hough transform is used to adaptively detect initial contours of the contourlet low-pass images in a multiresolution hierarchy. In this paper, the lumen and vessel wall structure of intravascular ultrasound image were roughly approximated as circulars; thus we may profit from this idea to use the Hough transform method to extract the two initial contours.

**The Hough Transform**

**Figure 2:**
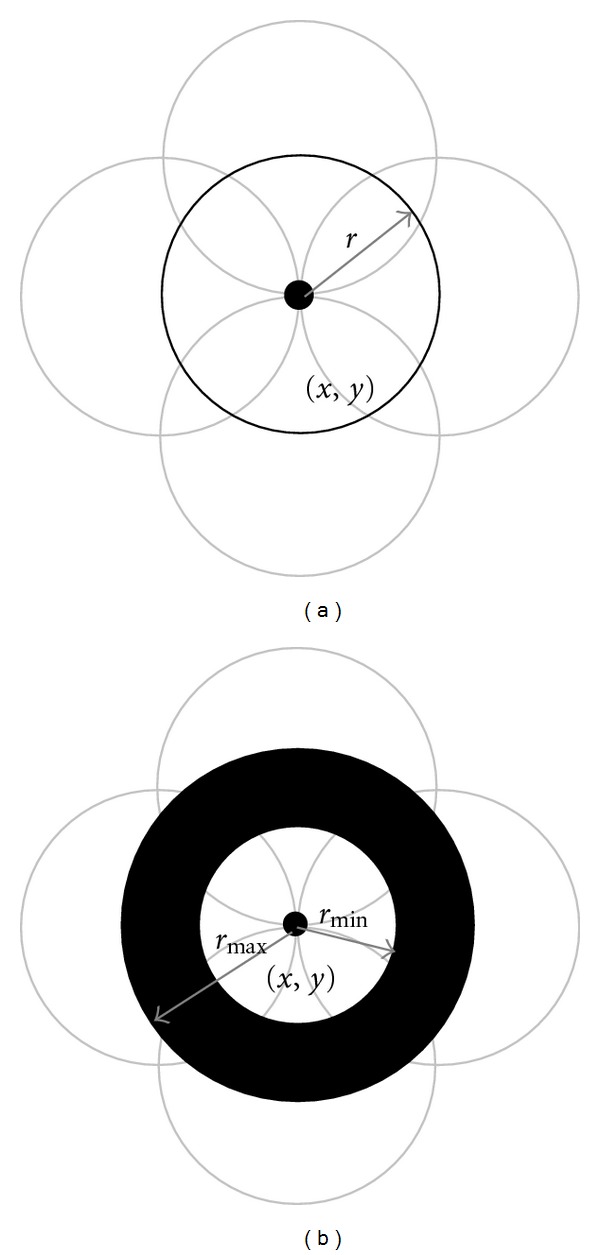
Idea of voting: (a) classical CHT, (b) CHT with multiple radii in one parameter space.

The Hough transform has been recognized as a very powerful tool for the detection of parametric curves, such as lines and circles, present in images. Tian et al. [13] noticed that the geometry of objects at eye images are close to circles, so they used the circular Hough transform (CHT) to deduce the radius and centre coordinates of the pupil and iris regions. Inspired by their work, we notice that the geometry of the lumen and media-adventitia contours tends to be circular. Thus, we may profit from this idea to use the circular Hough transform to extract the two initial contours in IVUS image. Firstly, an original image was transformed to edge map by calculating the first derivatives of intensity values. Then, votes are cast in an appropriately Hough space (Figure 2(a)) for the parameters of circles passing through each point. The parameters of the detected curves (circles) are the centre coordinates *x*_*c*_ and *y*_*c*_ and the radius *r*, which are able to define the equation of any circle as follows:

(11) $$x_{c}^{2}+y_{c}^{2}=r^{2}.$$

Accordingly, a maximum image point in the Hough space will correspond to the radius and centre coordinates of the circle best defined by the edge points. We will make use of the parabolic Hough transform to detect the circles, approximating the upper and lower contours of the vascular boundaries with parabolic arcs, which are defined as

(12) $$\begin{array}{cc} & {\left(\left(x-h_{j}\right)\sin\theta_{j}-\left(y-k_{j}\right)\cos\theta_{j}\right)}^{2}\\ & \;=a_{j}\left(\left(x-h_{j}\right)\cos\theta_{j}+\left(y-k_{j}\right)\sin\theta_{j}\right),\;j=1,\;2, \end{array}$$

where the constant *a*_*j*_ controls the curvature, (*h*_*j*_, *k*_*j*_) denotes the peak of the parabola, and *θ*_*j*_ is the angle of rotation relative to the *x*-axis.

The algorithm for circular Hough transform can be summarized to the following.

- Threshold original image to get a binary shape image.
- Extract just the edge points of the binary image using the Canny edge detection operator.
- Draw a circle with center in the edge point with radius *r*, and increment all coordinates that the perimeter of the circle passes through in an accumulator.
- Find one or several maxima in the accumulator.
- Map the found parameters (*a*_*j*_, *h*_*j*_, *k*_*j*_) corresponding to the maxima back to the original image.

**The Circular Hough Transform of Contourlet Low-Pass Bands**

The CHT algorithm is more accurate and robust when hardly visible circles can be detected and noise in an image does not affect the performance of the detection. In the case of IVUS images, the CHT is used in multiresolution hierarchy to gradually detect the two desired circles. As mentioned before, the contourlet low-pass components *a*_1_(*x*, *y*), *a*_2_(*x*, *y*),…, *a*_*J*_(*x*, *y*), can be regarded as multiresolution subband images. Each coarse image coefficient *a*_*j*_(*x*, *y*), 1 ≤ *j* ≤ *J*, is obtained by the low-pass filtering of a finer coefficient *a*_*j*−1_(*x*, *y*), and it is an approximate subimage of *a*_*j*−1_(*x*, *y*), with less speckle noise and fewer edge details. Then a coarser coefficient, *a*_*j*_(*x*, *y*), is feasible for computing the CHT algorithm, which is then used to detect the initial contours. When the multiresolution scale is decreased from *j* to *j* − 1, the CTH will be updated by a new subimage, *a*_*j*−1_(*x*, *y*), and a finer initialization will be produced. The CTH algorithm is used iteratively until the original *a*_0_(*x*, *y*) is initialized. The procedure for the multiresolution initialization based on the CHT is presented as follows.

(1) Start the analysis from level *j* = *J*. Define *I*_*j*_(*x*, *y*) as a coarse to fine IVUS image to be Initialized (*I*_*j*_(*x*, *y*) = *a*_*J*_(*x*, *y*)).
(2) Calculate gradient images along the vertical and horizontal direction and find the image edges using Canny operator.
(3) Compute the circular Hough transform to find the lumen contour. The result of executing CHT on image *I*_*j*_(*x*, *y*) produces a lot of circles that all of them are wrong except one circle that fits on lumen contour.
(4) Construct the 2D histogram of *I*_*j*_(*x*, *y*), and approximate the coordinate of the lumen centre *ac*(*x*_*ac*_, *y*_*ac*_). Then, choose a circle whose center is nearest to approximate lumen center rather than other circles. Euclidean distance can be used to calculate distance between the center of circles and approximate center of lumen that can be expressed as follows:
  (13) $$\mathrm{dist}=\sqrt{{\left(x_{cc}-x_{ac}\right)}^{2}+{\left(y_{cc}-y_{ac}\right)}^{2}},$$
  where *cc*(*x*_*cc*_, *y*_*cc*_) is coordinate of the circle center. After calculate distance, nearest circle found by CHT is fitted to lumen.
(5) Choose the outer vascular circle among the candidate circles whose center is nearest to the lumen center and having a maximum change in intensity. To make the searching of the optimal lumen and media-adventitia circles accurate, the parameters in (11) are limited to a certain range. First, for media-adventitia contour detection, we limit the distance between (*x*_*cc*_, *y*_*cc*_) and the center of the lumen, to *D*_*c*_, and *r* to a range from *r*_min⁡_ to *r*_max⁡_. Then, for lumen contour, *r*_max⁡_ is updated to half the radius of the circle found in media-adventitia contour.
(6) Update *I*_*j*−1_(*x*, *y*) by *a*_*j*−1_(*x*, *y*), and then return to step (2) to iteratively perform the algorithm; otherwise the optimal initialization ends here.

**Figure 3:**
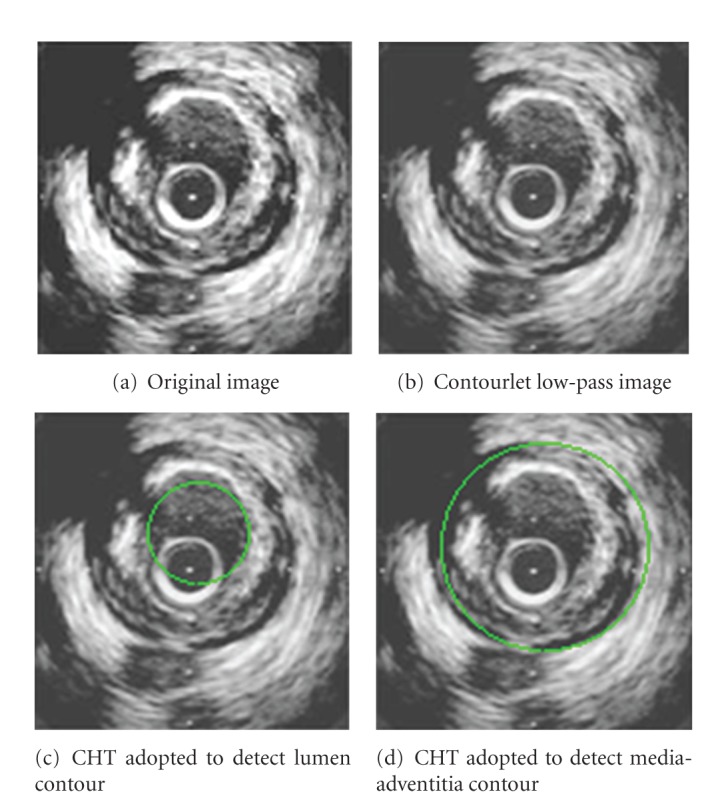
Initial contour extraction.

An example of the initial contours extraction is illustrated in Figure 3. The lumen boundary is extracted by the circular Hough transform as shown in Figure 3(b). Then the CHT is used to detect the initial media-adventitia boundary as shown in Figure 3(c).

#### 2.2.2. Final Contour Extraction

Contourlet low-pass components are low-frequency signals mainly reflecting the region of information of vascular tissue image. Contourlet band-pass components are relatively high-frequency signals mainly reflecting the organization of edges information. The band-pass subbands contain the directional information of the edges in IVUS image. Therefore, the final contour extraction of the lumen and media-adventitia boundaries is based on the band-pass directional subbands. The anisotropic diffusion filtering is used in each subband according to characteristics of the subband edges and noise. It reduces noise, while preserving and even strengthening useful edges. After the filtering of the subband signals, the contourlet reconstruction is utilized to obtain a filtered image, where the level-set evolution model is applied to delineate the two final contours.

The ultrasound images usually present a low signal-to-noise ratio and are affected by a form of multiplicative and locally correlated noise called speckle. Thus it is difficult to effectively remove the noise, as well as preserve useful edges in these kinds of images. As mentioned before, the contourlet transform decomposes an original image into a set of low-pass components and band-pass components. The band-pass bands can be regarded as multiresolution directional images from fine to coarse *c*_*j*,*k*_^*l*_*j*_^(*x*, *y*), *j* = 1, 2,…, *J*, *k* = 0, 1,…, 2^*l*^ − 1. Each band-pass band at scale *j* and direction *k* contains specific information about the edge and noise. Thus, it is worthwhile to use a new filtering technique based on the anisotropic diffusion (AD) of each subband to reduce the speckle effectively while preserving the edges.

The anisotropic diffusion (AD) is the more recent filtering procedure [11, 14]. It has been widely applied for a large number of applications in medical image processing field. The AD method uses iteratively nonlinear evolution partial differential equations (PDE) to improve an input image quality:

(14) $$\frac{\partial I}{\partial t}=\mathrm{div}\left[c\left(||\nabla I||\right)\cdot \nabla I\right],\;I\left(t=0\right)=I_{0},$$

where div is the divergence operator, *I*_0_ is the input image, and *c*(||∇*I*||) is the diffusion function, which can be given by

(15) $$c\left(||\nabla I||\right)=\exp\;\left[1+{\left(\frac{||\nabla I||}{g}\right)}^{2}\right],$$

where *g* is the gradient threshold.

Here, the AD filtering is extended to the mutiresolution contourlet domain, and the directional subcomponents, other than the original image, are filtered by the AD. Thus, the image *I*_0_ in (14) is substituted with *c*_*j*,*k*_^*l*_*j*_^(*x*, *y*), and then the noise in *c*_*j*,*k*_^*l*_*j*_^(*x*, *y*) will be removed by the AD while edges are enhanced to obtain *c*_*j*,*k*_′(*x*, *y*). At each scale *j* = 1,2,…, *J*, the directionally filtered subbands *c*_*j*,*k*_′(*x*, *y*) are pooled together to get cumulative band-pass signal *c*_*j*_′(*x*, *y*):

(16) $$c_{j}^{\prime }\left(x,y\right)=\overset{2^{lj}}{\underset{k=0}{\sum }}c_{j,k}^{\prime }\left(x,y\right).$$

*c*_*j*_′(*x*, *y*), along with the low-pass image *a*_*J*_(*x*, *y*), is used to obtain the final filtered image *I*′ by the LP reconstruction.

Then the new level-set segmentation model (CLSM) is applied to the image *I*′. The two initial contours deform under the action of *internal *and *external* energies until converge to the final contours.

## 3. Results

The proposed method was validated with the traditional level-set segmentation method on both simulated and real IVUS images. The parameters of this algorithm were set as follows: the level number of the LP decomposition was *J* = 3, and the level numbers of the DFB decomposition were *K* = 2, 2, and 3 at LP levels 1–3; respectively, the radii range value for the CHT is (*r*_min⁡_, *r*_max⁡_) = (15,40) and *D*_*c*_ = 25. In experiments, we first make quantitative comparison of the visual observation between a traditional level-set segmentation method such as that proposed by Li et al. [12] and our proposed method. Then we perform a qualitative comparison between the results of the two methods.

### 3.1. Simulated Images

**Figure 4:**
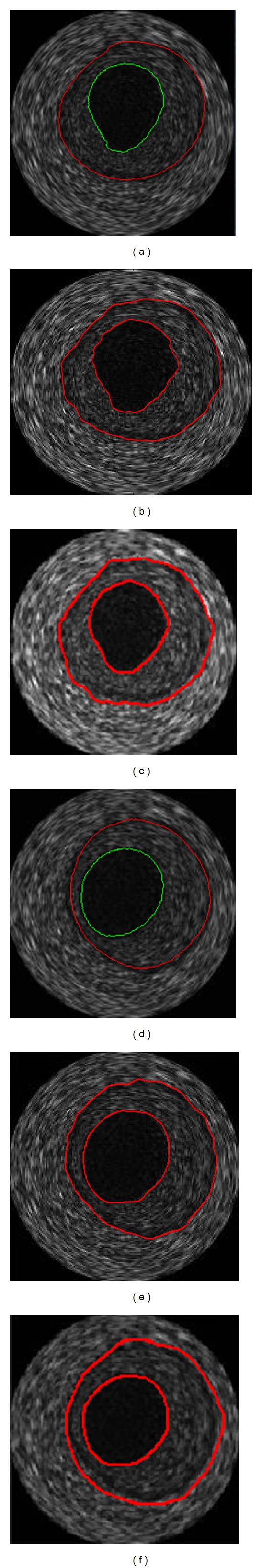
Two examples of simulated images segmentation results. ((a), (d)) Real boundaries (ground truth), ((b), (e)) traditional method, and ((c), (f)) the proposed method.

Simulated IVUS images were conducted to evaluate algorithms of vessel boundaries detection. Since the exact geometry of simulated images is known, the calculation of the segmentation accuracy is obtained directly. The generation of simulated IVUS images was based on a polar image-formation model [15]. To make the simulation of IVUS data more realistic, the ring-down and guide-wire artifacts were merged in this model. Typical results on simulated IVUS images for each segmentation method were displayed in Figure 4.

These results demonstrated that detected boundaries for the two methods were very close to the vessel wall structures. They also showed that the media-adventitia border was smoother with the CLSM method than with the traditional method. However, the lumen, which often has strong edges, was detected with sufficient details.

To quantify the segmentation accuracy, mean distance error (MD) and relative mean distance error (RMD) [16] between the manually traced contours and the two segmentation results were calculated. The MD and RMD are called metric errors, evaluating the distance between the automatically detected contour and the ground truth curve. Moreover, to assess the repeatability of the segmentation results for each method under different initializations, the mean distance error and relative mean distance error between results were calculated.

Let *p* be a contour point detected by proposed automated method and *q* the nearest point in the traditional method. The distance between *p* and *q* (i.e., the minimum distance between the discrete point *p* and the ground truth contour) is *D*(*p*). The relative distance error is *RD*(*p*) = *D*(*p*)/*d*(*q*, *O*), where *O* is the center of the ground truth contour and *d*(*q*, *O*) is the distance between *q* and *O*. Then the MD and RMD are defined as follows:

(17) $$\begin{array}{c} \text{MD}={\text{mean}}_{p}\left(D\left(p\right)\right),\\ \text{RMD}={\text{mean}}_{p}\left(RD\left(p\right)\right)\times 100\%. \end{array}$$

Table 1 shows the quantitative comparison of the results for 20 simulated images. For both lumen and media-adventitia borders, the two quantitative indicators are better with the proposed method than with traditional method. Compared with traditional boundary segmentation results, we can see that the indices MD and RMD of our method were increased by 7.32 pixels and 8.76%, respectively.

### 3.2. Real IVUS Images

The in vivo IVUS images used in this study were acquired at the Department of Cardiology, Errabta Hospital of Tunis, Tunisia. Data were acquired with a Jomed equipment using a 312F 30-MHz catheter (In-vision gold, Helsingborg, Sweden). IVUS sequence images were captured from 7 patients through the pullback of the transducer in coronaries arteries and were digitized on 384 × 384 pixels, then stored using the DICOM standard. The acquisition was done at a 10 images/sec frame rate, and the transducer pull-back velocity was set to 1 mm/sec. Acquisition parameters were set by physicians to optimize the image quality.

**Qualitative Comparison**

**Figure 5:**
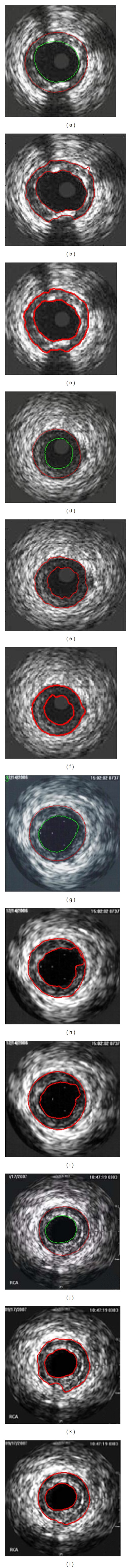
Three examples of real images segmentation results. ((a), (d), (g), (j)) Real boundaries (ground truth), ((b), (e), (h), (k)) traditional level-set model, and ((c), (f), (i), (l)) the proposed method.

For examples of the segmentation results for the two methods are shown in Figure 5. The lumen and media-adventitia detected boundaries are presented for four different in vivo IVUS images. A qualitative analysis of the contourlet-based and traditional level-set segmentation revealed that detected contours were very close to the two vessel boundaries. In the first example shown in Figures 5(b) and 5(c), it was demonstrated that the media-adventitia boundaries are closer to the ground truth than CLSM method than with traditional method, because speckles have been reduced with contourlet transform. However, the lumen was identified correctly by the two methods because it has strong edges. In the second example, the two borders detected by the traditional method appeared contracted (Figure 5(e)), because this IVUS image was more contaminated with noise. In contrast, the two boundaries detected by our proposed method (Figure 5(f)) were close to manual traced boundaries. In the third and forth examples, the boundaries detected by our proposed method (Figures 5(i) and 5(l)) were satisfactory and close to the ground truth (Figures 5(d) and 5(g)), where the borders detected by the traditional method (Figures 5(h) and 5(k)) were distributed by the speckle noise.

**Quantitative Comparison**

Table 2 shows the quantitative comparison of the segmentation results of 30 real images. Two indices, the mean distance error (MD) and the relative mean distance error (RMD), were calculated between the manually traced and the automatic detected boundaries. For the media-adventitia boundary, these two indices are better with the proposed method than with the traditional method. However, higher indicators were obtained on lumen boundary with the traditional method because the blood frontier in nontransformed images produces bright echoes for which the gradient information is more significant. Compared with results of traditional method, MD and RMD of the proposed method were improved by 5.30 pixels and 7.45%, respectively.

## 4. Conclusions

This paper presents a new IVUS plaque image segmentation method based on level-set model and contourlet transform. In traditional methods of level-set model, there are two problems: initialization and robustness. The contourlet transform provides some improvements. The Hough transform is adopted in multiresolution low-pass bands to yield the initial contours of lumen border and media-adventitia borders. The anisotropic diffusion is then utilized in band-pass directional subbands to suppress the noise as well as preserve vascular boundaries, and the contour evolution in the level-set functions is used to obtain final contours. Experimental results on 20 simulated images and 30 in vivo demonstrated the usefulness of the method to automatically and accurately extract the two boundaries of the blood vessels, and results demonstrate that the mean distance error and the relative mean distance error have increased by 5.30 pixels and 7.45%, respectively, as compared with those of the traditional level-set model.

It was demonstrated that the segmentation results of lumen boundary of simulated and real images are better than the media-adventitia boundary. The lumen, which can usually have rougher and obvious surface, was accurately detected with sufficient details. However, the accuracy of media-adventitia boundary detection depends on the shape and position of the initial contour in the image. Therefore, when the initial contour is close to the true contour, the final segmentation result is better.

## Figures and Tables

**Table 1:** Quantitative comparison of the results segmented from simulated images.

Borders	Method	MD	RMD
Lumen boundary	Traditional method	8.582	12.38%
	Proposed method	5.653	8.76%
Media-adventitia boundary	Traditional methods	11.09	16.01%
	Proposed method	7.323	11.31%

**Table 2:** Quantitative comparison of results segmented from real images.

Borders	Method	MD	RMD
Lumen boundary	Traditional method	7.7345	11.21%
	Proposed method	4.5627	6.30%
Media-adventitia boundary	Traditional methods	9.1550	13.27%
	Proposed method	5.3996	7.46%
